# Barriers and Facilitators to Advance Care Panning Among Veterans Aging With HIV

**DOI:** 10.1093/geroni/igab046.1638

**Published:** 2021-12-17

**Authors:** Emily Pinto Taylor, Sean Halpin, Vincent Marconi, Amy Justice, Theodore Johnson II, D Keith McInnes, Molly Perkins

**Affiliations:** 1 Emory University School of Medicine, Atlanta, Georgia, United States; 2 University of Georgia, Decatur, Georgia, United States; 3 Division of Infectious Diseases, Emory University School of Medicine, Atlanta, Georgia, United States; 4 West Haven VA Medical Center, West Haven, Connecticut, United States; 5 Boston University School of Public Health, Boston, Massachusetts, United States; 6 Emory University of Medicine, Atlanta, Georgia, United States

## Abstract

Advance care planning (ACP) and hospice services are underutilized by patients living with HIV (PWH). Little is known about how older PWH approach ACP; the purpose of this qualitative study was to understand barriers and facilitators to ACP within the context of the patient-clinician relationship. Data are from a larger multimethod study designed to understand social determinants of health (SDH) that shape the lives and healthcare experiences of veterans aging with HIV. The sample includes 25 veterans from the Veterans Aging Cohort Study (VACS) recruited from an urban VA medical center. Semi-structured interviews were performed and analyzed using thematic analysis. Less than half of participants reported engaging in ACP. Key barriers to ACP include: fragile social ties, distrust of the healthcare system, and fear of disclosure and discrimination. We offer several recommendations for clinicians to engage in these conversations successfully and highlight the importance of considering SDH when designing interventions.

